# Antisense oligonucleotide treatment rescues UBE3A expression and multiple phenotypes of an Angelman syndrome mouse model

**DOI:** 10.1172/jci.insight.145991

**Published:** 2021-08-09

**Authors:** Claudia Milazzo, Edwin J. Mientjes, Ilse Wallaard, Søren Vestergaard Rasmussen, Kamille Dumong Erichsen, Tejaswini Kakunuri, A.S. Elise van der Sman, Thomas Kremer, Meghan T. Miller, Marius C. Hoener, Ype Elgersma

**Affiliations:** 1 Departments of Clinical Genetics and Neuroscience and; 2 ENCORE Expertise Center for Neurodevelopmental Disorders, Erasmus MC, Rotterdam, Netherlands.; 3 Therapeutic Modalities, Roche Innovation Center Copenhagen, F. Hoffmann-La Roche Ltd., Horsholm, Denmark.; 4 Neuroscience and Rare Diseases Discovery & Translational Area, Roche Innovation Center Basel, F. Hoffmann-La Roche Ltd., Basel, Switzerland.

**Keywords:** Development, Neuroscience, Mouse models, Neurodevelopment, Neurological disorders

## Abstract

Angelman syndrome (AS) is a severe neurodevelopmental disorder for which only symptomatic treatment with limited benefits is available. AS is caused by mutations affecting the maternally inherited ubiquitin protein ligase E3A (*UBE3A*) gene. Previous studies showed that the silenced paternal *Ube3a* gene can be activated by targeting the antisense *Ube3a-ATS* transcript. We investigated antisense oligonucleotide–induced (ASO-induced) *Ube3a-ATS* degradation and its ability to induce UBE3A reinstatement and rescue of AS phenotypes in an established *Ube3a* mouse model. We found that a single intracerebroventricular injection of ASOs at postnatal day 1 (P1) or P21 in AS mice resulted in potent and specific UBE3A reinstatement in the brain, with levels up to 74% of WT levels in the cortex and a full rescue of sensitivity to audiogenic seizures. AS mice treated with ASO at P1 also showed rescue of established AS phenotypes, such as open field and forced swim test behaviors, and significant improvement on the reversed rotarod. Hippocampal plasticity of treated AS mice was comparable to WT but not significantly different from PBS-treated AS mice. No rescue was observed for the marble burying and nest building phenotypes. Our findings highlight the promise of ASO-mediated reactivation of *UBE3A* as a disease-modifying treatment for AS.

## Introduction

Angelman syndrome (AS) is a severe neurodevelopmental disorder affecting approximately 1:20,000 individuals (1). It is characterized by severe intellectual disability, motor coordination deficits, sleeping abnormalities, epilepsy, speech impairment, and behaviors associated with autism spectrum disorder (2). Currently, only symptomatic treatments are available to treat AS; therefore, it is of great importance to develop a disease-modifying treatment.

**Figure 1:**
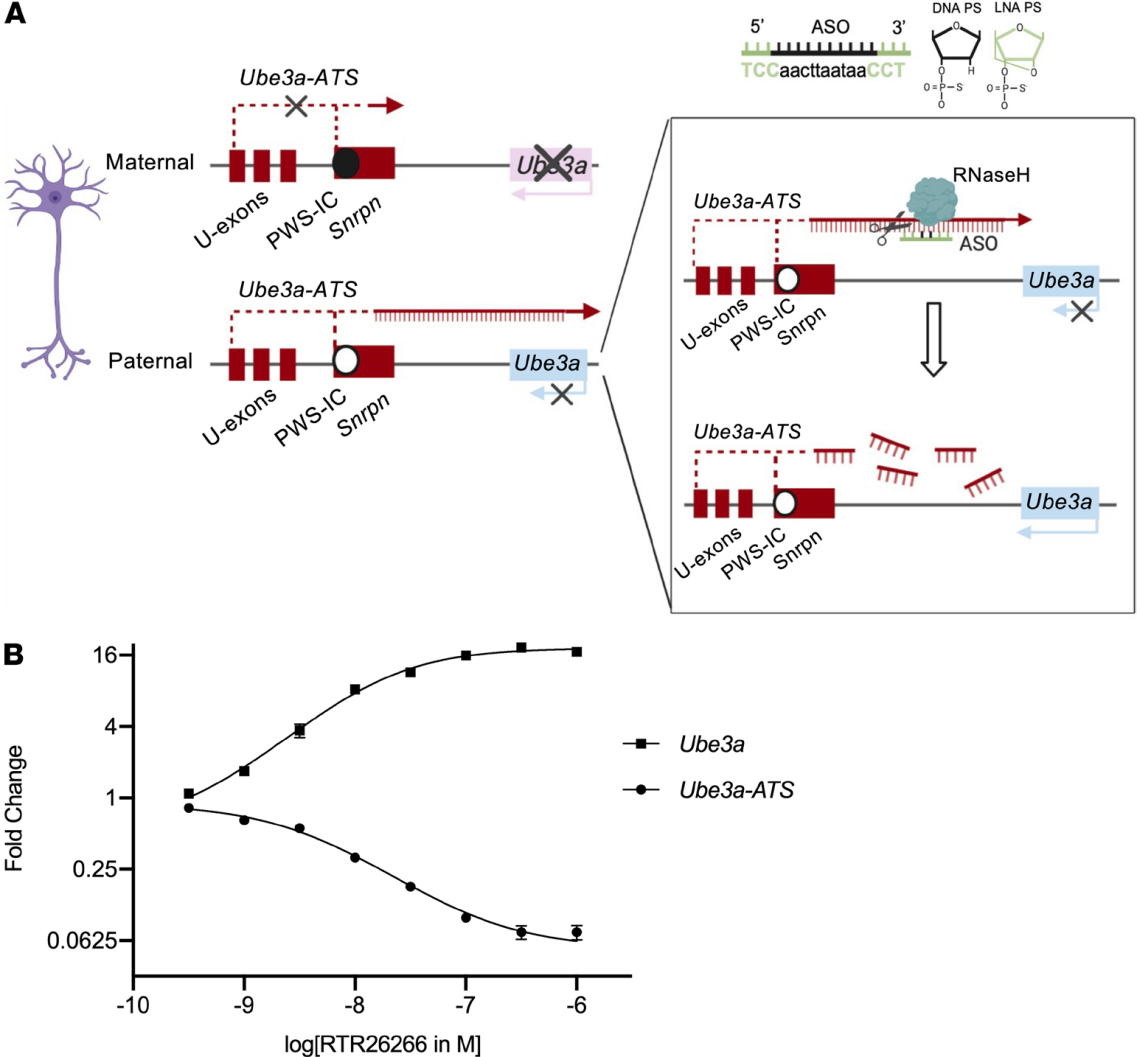
Paternal *Ube3a* silencing and ASO strategy to restore its expression in mouse neurons. (**A**) Schematic representation of the mouse *Ube3a* genomic locus. Analogous to human, transcription of the paternal *Ube3a-ATS* from the same promoters encoding *Snrpn*, upstream of the *PWS-IC* (black filled circle), interferes with *Ube3a* transcription. For the knockdown of the *Ube3a-ATS*, a 3-10-3 gapmer antisense oligonucleotide (ASO) with phosphorothioate (PS) (black) and locked nucleic acid (LNA) modifications (green) for 3 nucleotides flanking the DNA core (black) was used (sequence indicated above). Upon RNaseH cleavage at the RNA/DNA hybrid site, the *Ube3a-ATS* was degraded; as a result *Ube3a* was unsilenced. (**B**) Concentration response curve of RTR26266 (M) in AS mouse neurons measuring both *Ube3a-ATS* and *Ube3a* mRNA expression.

AS is typically caused by large genomic deletions of the 15q11-13 region, affecting multiple genes, of which loss of the maternal copy of the ubiquitin protein ligase E3A (*UBE3A*) gene is causal for AS (3, 4). UBE3A is an E3 ubiquitin-protein ligase involved in the ubiquitin-proteasome system and is important for normal neurodevelopment and synaptic function (5). In neurons, the paternal *UBE3A* allele is silenced as a result of transcriptional interference by the long noncoding *UBE3A-ATS* (antisense) transcript (6–8). In both humans and mice, the *UBE3A-ATS* transcript originates from the same precursor encoding small nuclear ribonucleoprotein N (*SNRPN*), which has multiple upstream promoters in the Prader-Willi syndrome imprinting center (*PWS-IC*) and upstream of the U exons where it initiates, and it ultimately overlaps with the *UBE3A* gene (Figure 1A) (9). The combination of both paternal *UBE3A* silencing and loss of maternal *UBE3A* expression due to mutations in the maternal allele in AS patients results in the complete loss of neuronal UBE3A protein expression in AS patients. In mice, it has previously been shown that the *Ube3a-ATS* transcript can be effectively targeted to restore paternal *Ube3a* expression (Figure 1A). Meng and colleagues showed that transcription inhibition of the *Ube3a-ATS* transcript, either by deletion of the genomic area around its promoter or by insertion of a transcriptional stop cassette to induce premature termination, leads to paternal *Ube3a* expression (9, 10).

ASOs are single-stranded nucleic acid molecules able to bind RNA through Watson-Crick base pairing and lead to the degradation of the targeted RNA by recruiting the RNaseH endonuclease. Therefore, ASOs are especially suited for specific and effective knockdown of the *Ube3a-ATS* transcript (11). Since their discovery in 1978, ASOs have been chemically modified to increase their efficacy, half-life, and specificity for the RNA target (12). Currently, there are many chemistries that can be used to modify ASOs. Each chemistry offers different advantages and disadvantages and can be more or less suited to target a specific sequence (11). Very commonly used are the PS, 2′-O-methoxyethyl (2′-MOE), and LNA modifications. The PS chemistry is commonly used as a backbone for the ASOs because it confers nuclease resistance and increased cellular uptake (11). The 2′-MOE and the LNA chemical modifications can provide resistance to nucleases and increased specificity for the target but inhibit RNaseH recognition of the heteroduplex (11). Hence, ASOs are designed with a gapmer configuration characterized by a DNA core that enables DNA/RNA heteroduplex recognition by RNaseH and chemically modified RNA wings (13). ASOs’ safety and efficacy have been proved for a number of disorders, and promising safety and pharmacodynamic data strongly suggest that ASOs can be applied to severe central nervous system pathologies (e.g., Huntington disease, amyotrophic lateral sclerosis, and spinal muscular atrophy) (14).

The potential of ASOs to treat AS has been shown in a pioneering study that used 2′-MOE ASOs to break down the *Ube3a-ATS* and reinstate *Ube3a* expression in adult mice (15). However, this treatment was largely insufficient to rescue behavioral AS phenotypes in vivo (15). Failure of phenotypic reinstatement in adult AS mice might have resulted from the level of UBE3A reinstatement achieved (35% in cortex and hippocampus) or because earlier therapeutic intervention is needed. Previous studies of our lab showed that reinstatement of UBE3A expression during embryonic development or around birth rescues all AS phenotypes whereas intervention in adult mice is not effective (16, 17). Furthermore, deletion of *Ube3a* in mice at 3 weeks of age results in only very mild behavioral deficits, emphasizing the necessity of UBE3A expression during early postnatal brain development (18). Hence, in the present study, we treated young AS mice with a PS and LNA modified gapmer ASO and subjected them to biochemical, electrophysiological, and behavioral analyses.

## Results

### Selection of the most effective ASO for targeting and knockdown of the Ube3a-ATS transcript.

To identify a potent ASO for the reinstatement of *Ube3a* in vivo, we designed 192 ASOs targeting the *Ube3a-ATS* transcript in a region downstream of the *Ube3a* gene. A small set of efficacious compounds, showing 15- to 20-fold increase in *Ube3a* expression, was identified in mouse primary cortical neurons, and 3 of them were selected for further in vivo testing. Two of the 3 ASOs injected at P1 given at an initial testing dose of 11 and 22 μg led to overt signs of toxicity in mice (weight loss, trembling body, rough fur). Using a lower dose prevented any sign of toxicity but resulted in limited UBE3A reinstatement (data not shown). The remaining ASO, RTR26266, showed a potent *Ube3a-ATS* knockdown (EC_50_ = 4 nM) and *Ube3a* upregulation (EC_50_ = 16 nM) in vitro (Figure 1B) and was well tolerated in vivo by a single intracerebroventricular (ICV) injection at 22 μg and 91 μg for P1 and P21 mice, respectively.

### ASO treatment of P1 AS mice efficiently restores brain-wide UBE3A protein levels.

**Figure 2:**
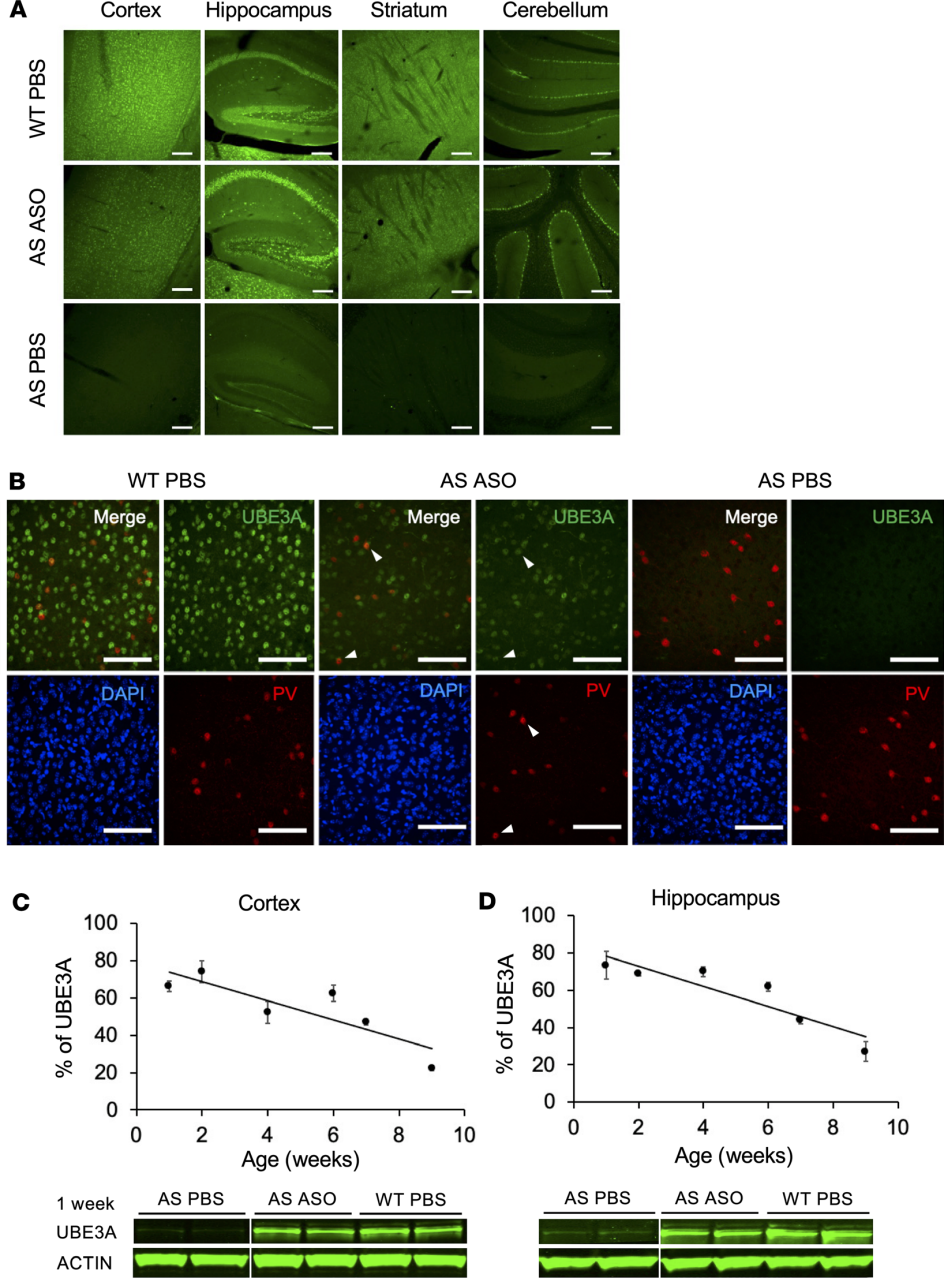
P1 injection with ASO restores UBE3A expression in the brains of AS mice. (**A** and **B**) Immunostaining at 4 weeks postinjection. (**A**) Brains (*n* = 2) of WT-PBS, AS-ASO, and AS-PBS injected mice were stained for UBE3A (green), showing widespread UBE3A reinstatement in ASO-treated AS mice. (**B**) Cortices (*n* = 2) of WT-PBS, AS-ASO, and AS-PBS injected mice stained for UBE3A (green) and parvalbumin (PV) (red) and counterstained for DAPI (blue), indicating that PV cells are positive for UBE3A. (**C** and **D**) Quantification of UBE3A expression in the cortex and hippocampus of ASO-treated AS mice and AS-PBS and WT littermates at different time points after P1 injection. Age is indicated in weeks on the *x* axis, and percentage of UBE3A in ASO-treated AS mice compared with WT mice is indicated on the *y* axis. One week after injection, UBE3A could be detected at 100 kDa. Actin, used as loading control, was detected at 45 kDa. The white vertical lines separating bands indicate noncontiguous lanes that were run on the same gel. Week 1: AS-ASO, *n* = 14; AS-PBS, *n* = 2; WT-PBS, *n* = 9. Week 2: AS-ASO, *n* = 3; WT-PBS, *n* = 3. Week 4: AS-ASO, *n* = 3; WT-PBS, *n* = 3. Week 6: AS-ASO, *n* = 2; WT-PBS, *n* = 3. Week 7: AS-ASO, *n* = 4; WT-PBS, *n* = 3. Week 9: cortex, AS-ASO, *n* = 7; WT-PBS, *n* = 4. Hippocampus, AS-ASO, *n* = 7; WT-PBS, *n* = 5. Scale bars: 200 μm (**A**), 100 μm (**B**). Data are represented as means ± SEM.

Since the optimal treatment window for restoring UBE3A expression in AS mice was shown to be around birth (16, 17), injections of ASO RTR26266 to reinstate *Ube3a* in vivo were performed in neonatal (P1) *Ube3a^m–/p+^* (AS) mice (19). A single dose of 22 μg of ASO was injected ICV. AS and WT littermates injected with PBS were used as controls. Four weeks after treatment, a strong reinstatement of UBE3A was observed throughout the whole brain of ASO-treated AS mice (Figure 2A and Supplemental Figure 1A; supplemental material available online with this article; https://doi.org/10.1172/jci.insight.145991DS1). Although the overall distribution of UBE3A expression was similar to WT mice, a notable exception is the dentate gyrus, a brain area that shows continuing neurogenesis (20, 21). Staining with parvalbumin, a marker of GABAergic neurons, showed that UBE3A reinstatement in ASO-treated AS mice was not limited to excitatory neurons (Figure 2B and Supplemental Figure 1, B–D). To evaluate the long-term effect of a single ASO injection, the cortex and hippocampus of ASO-treated AS mice were analyzed by Western blot 1, 2, 4, 6, 7, and 9 weeks after P1 injection. In both cortex and hippocampus of ASO-treated AS mice, UBE3A reached peak levels of ±70% 1 week after injection (Figure 2, C and D; and Supplemental Figure 2, A and B). In both areas of the brain, UBE3A expression levels slowly declined to 22%–27% in the following 8 weeks (Figure 2, C and D; and Supplemental Figure 2, A and B). A decrease of UBE3A expression in time was expected due to the initial expansion of the mouse brain, which reaches 91% of its total weight only at P21, causing a dilution of the ASO (21), and due to the half-life of the ASO itself. Given that the UBE3A protein has a half-life of approximately 4 days (22), and that throughout the 9 weeks following ASO injection we observed a linear rather than exponential reduction of UBE3A levels, the ASO must have remained partially active throughout that period (Figure 2, C and D).

### ASO treatment of P1 AS mice rescues multiple AS mouse phenotypes.

**Figure 3:**
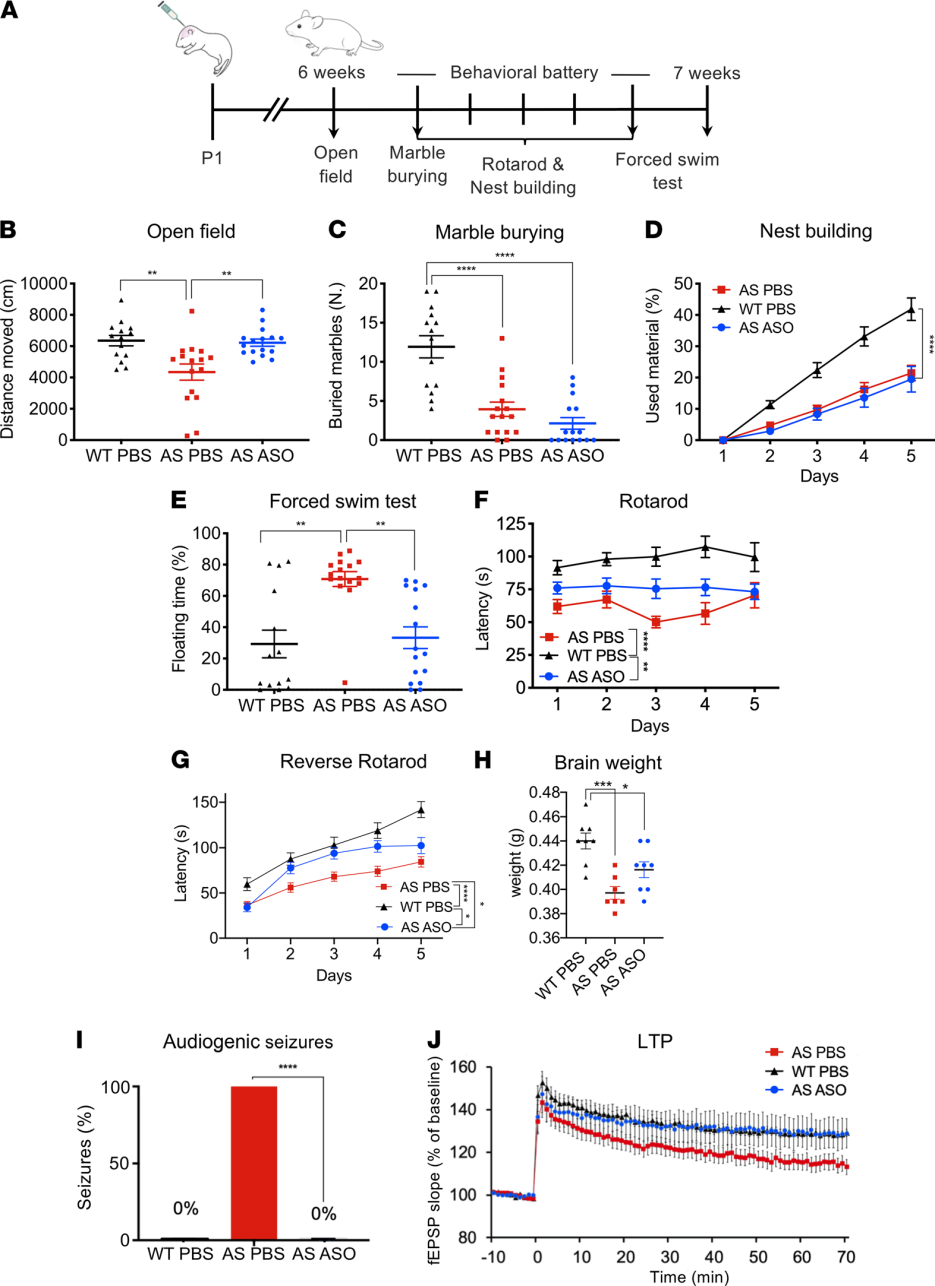
ICV injection of ASO at P1 rescues specific AS phenotypes. (**A**) Schematic representation of ASO treatment and timeline for behavioral testing. (**B**–**F**) Behavioral battery performed with WT-PBS (black, *n* = 14), AS-PBS (red, *n* = 16), and AS mice injected with ASO (blue, *n* = 16) in the open field, marble burying, nest building, forced swim tests, and accelerating rotarod. One-way or 2-way ANOVA was performed, with treatment plus genotype as independent variable for statistical analysis. (**G**) A new cohort of 6-week-old AS-PBS (*n* = 30), WT-PBS (*n* = 19), and AS-ASO (*n* = 17) mice was used for the reverse rotarod. A 2-way ANOVA was performed, with treatment plus genotype as independent variable for statistical analysis. (**H**) Brain weight of 7-week-old AS-PBS (*n* = 7), WT-PBS (*n* = 8), and AS-ASO (*n* = 8) mice. One-way ANOVA was performed, with treatment plus genotype as independent variable for statistical analysis. (**I**) Audiogenic seizures were rescued in P1-ASO–treated *Ube3a^–/+^* mice: susceptibility to clonic-tonic seizures was visually observed in AS mice (*n* = 6) and not observed in ASO-treated mice (*n* = 10) or WT mice (*n* = 7). Fisher exact test was used as the statistical test. (**J**) LTP in AS mice treated with ASO showed a trend similar to WT mice. Both gave a higher response than AS mice, but this difference was not significant. Two-way ANOVA, followed by Bonferroni’s post hoc test, was used as the statistical test, with treatment and genotype as independent variables. Number of slices/mouse measured: AS-PBS (*n* = 13/6); AS-ASO (*n* = 16/8); WT-PBS (*n* = 13/6). Data represented as mean ± SEM. *P* values represent the significance level for the parameter and are displayed as stars in the figure: not shown if *P* > 0.05, *P ≤ 0.05, **P ≤ 0.01, ***P ≤ 0.001, ****P ≤ 0.0001. fEPSP, field excitatory postsynaptic potential.

Encouraged by the efficacy of the selected ASO to reinstate UBE3A expression, we next evaluated whether a single ASO injection was sufficient to rescue behavioral deficits. Six weeks after P1 injection of the ASO in F1 hybrid 129S2-C57BL/6J *Ube3a^m–/p+^* mice, we tested the rescue of behavioral deficits using a well-established behavioral testing battery (22). However, to ensure testing was performed when UBE3A levels are still high, we modified this highly standardized protocol in order to perform all behavioral assays over a single week (Figure 3A).

Behavioral paradigms to test repetitive and perseverative behaviors, such as marble burying, or innate behaviors, such as nest building, showed no significant difference when comparing AS mice injected with ASO or PBS (Figure 3, C and D; see Supplemental Table 1 for all statistics). In contrast, anxiety, as assessed using the open field task, showed that the reduced distance AS animals moved was fully rescued in ASO-treated AS mice (Figure 3B). Performance of ASO-treated AS mice in the forced swim test was also significantly improved and comparable to WT mice (Figure 3E). Motor coordination deficiency was tested by assessing the performance on the accelerating rotarod. Until training day 4, AS mice injected with the ASO performed better than AS untreated mice, but there was no overall significant improvement in ASO-treated AS mice compared with the untreated mice over the full 5 days (Figure 3F).

In contrast to our previously published data (23), none of the mice showed significant motor learning over time. The difference with our previous data is that these mice were younger and single-housed and that all testing was compressed to a single week. This may have increased anxiety levels and impaired performance on the rotarod. To address this issue, we generated a new cohort of F1 hybrid 129S2-C57BL/6J *Ube3a^m–/p+^* mice to reinvestigate motor coordination performance. This new cohort of mice was group-housed, handled longer, and not tested in other behavioral tests. Moreover, we chose to use the reverse rotarod (in which the mice have to learn to walk backward), as this test shows larger differences between WT and AS mice (17). Indeed, a strong phenotype was observed between PBS-treated AS and WT mice, and both groups showed significant improvement over training sessions (Figure 3G). Moreover, the performance of ASO-treated AS mice in the reverse rotarod was significantly better compared with PBS-treated AS mice (Figure 3G). However, this phenotype was not completely rescued upon ASO injection, as ASO-treated AS mice performed similarly to PBS-treated WT mice from day 2 to 4, but on day 5 the 2 groups differed in performance, resulting in an overall significant difference between WT and ASO-treated AS mice in the reverse rotarod task (Figure 3G).

At 7 weeks of age, a subset of this cohort of mice was used to evaluate the extent to which a single injection of ASO at P1 rescued specific anatomical phenotypes of AS, such as increased body weight and microcephaly (23, 24). No difference in body weight was detected at 7 weeks of age (Supplemental Figure 3, A and B). Brain weight analysis showed a significant difference between PBS-treated AS and WT mice. Although ASO-treated AS mice showed a notable trend toward reduced microcephaly, this improvement did not reach significance (Figure 3H).

Next, we tested whether ASO injection can rescue epilepsy, one of the major symptoms of AS. *Ube3a^m−/p+^* mice in the 129S2 background were tested for audiogenic seizure susceptibility 6 weeks after P1 injection. Although all the AS animals injected with PBS showed seizures, none of the ASO-treated mice showed seizures (Figure 3I).

Last, we investigated whether hippocampal plasticity in P1 ASO-treated AS mice could be rescued. Long-term potentiation (LTP) was evoked by using the 10-theta burst protocol in hippocampal slices of mice that were sacrificed at 7 weeks of age. AS mice that received a single ASO injection at P1 were indistinguishable from WT mice (Figure 3J). Although LTP in PBS-treated AS mice was reduced to the commonly observed 50% of WT levels, this difference did not reach significance, due to the rather large standard deviation (Figure 3J).

### ICV injection of ASO at P21 also rescues epilepsy.

**Figure 4:**
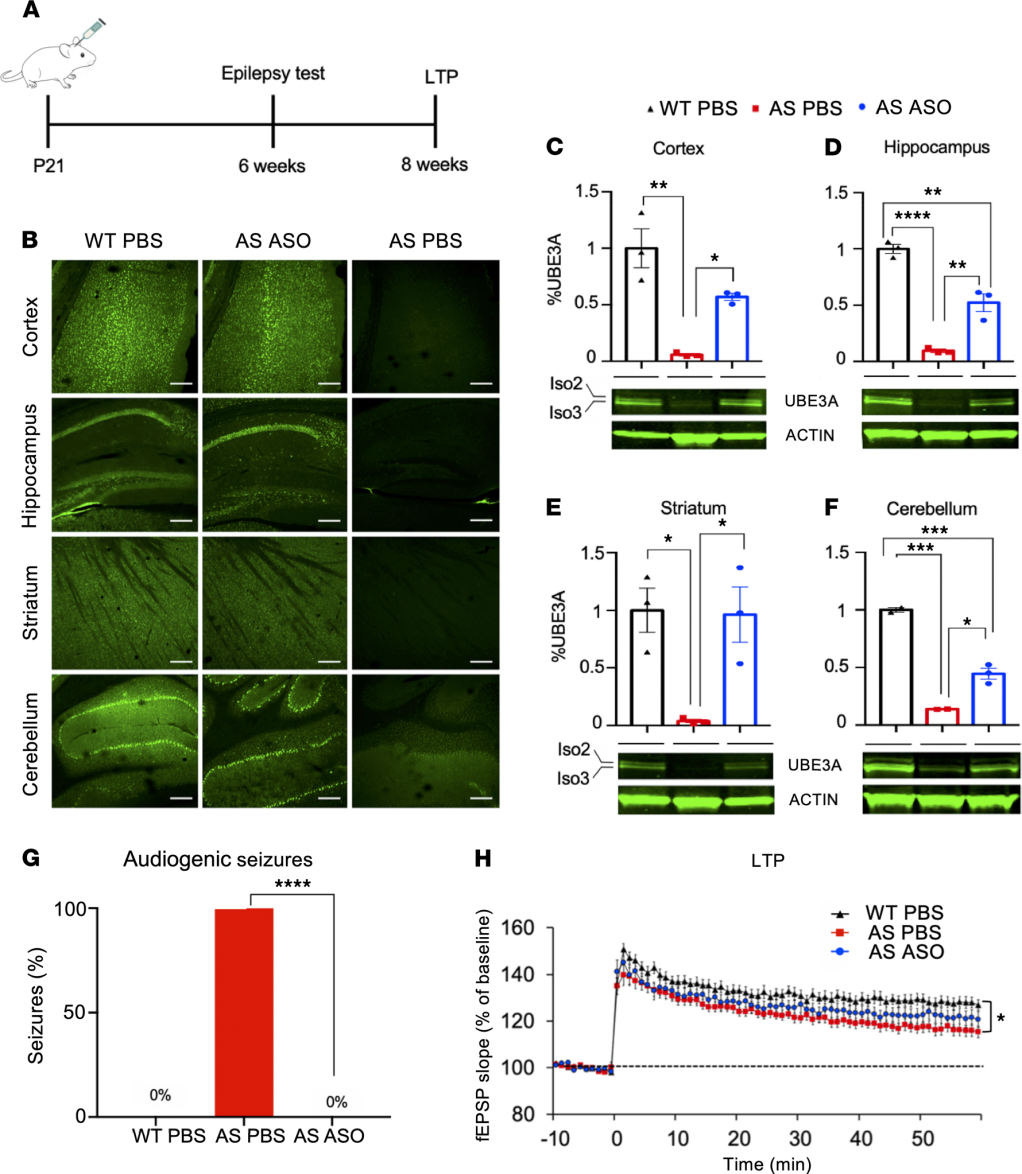
P21 injection with ASO successfully restores UBE3A expression in AS mice and rescues epilepsy and hippocampal plasticity. (**A**) Schematic representation of the timeline for ASO treatment, epilepsy testing, and LTP measurement. (**B**) Three weeks postinjection (*n* = 2) the brains of WT-PBS, AS-ASO, and AS-PBS mice were stained for UBE3A (green). The brains of AS mice treated with ASO show widely distributed nuclear UBE3A compared with the untreated AS brain. (**C**–**F**) Western blots of lysates obtained 4 weeks after P21 injection of cortex, hippocampus, striatum, and cerebellum of AS mice treated with ASO (*n* = 3) and compared with age-matched WT controls and AS mice injected with PBS (*n* = 3). Two bands, representing the cytosolic and nuclear isoforms of UBE3A, can be detected at 100 kDa and ACTIN, here used as loading control, at 45 kDa. One-way ANOVA, followed by Tukey’s post hoc test, was used as statistical test. (**G**) Susceptibility to seizures visually observed in AS mice (*n* = 10) is rescued when treated with ASO at P21 (*n* = 13). Seizures are also absent in WT mice (*n* = 9). Fisher exact test was used as statistical test. (**H**) LTP measurement in AS mice treated with ASO (blue line), WT mice (black line), and AS mice (red line). Two-way ANOVA, followed by Bonferroni’s correction, was used as the statistical test. Number of slices used/mouse: AS-PBS (*n* = 26/5); AS-ASO (*n* = 27/7); WT-PBS (*n* = 23/4). Scale bars: 200 μm (**B**). Data are represented as means ± SEM. *P* values represent the significance level for the parameter and are displayed as stars in the figure: not shown if *P* > 0.05, *P ≤ 0.05, **P ≤ 0.01, ***P ≤ 0.001, ****P ≤ 0.0001.

Given the very effective rescue of UBE3A protein levels upon P1 ASO injection, we investigated whether administration of the ASO in juvenile (P21) mice (129S2 background) would be equally effective in reinstating UBE3A protein levels and rescuing epilepsy (Figure 4A). Similar to what was observed after P1 injection, UBE3A protein levels were restored throughout the brains of P21 ASO-treated AS mice in multiple cell types (Figure 4B and Supplemental Figure 4, A–D). Four weeks after P21 injections, we observed, by Western blot analysis, that ASO-treated AS mice maintained a strong expression of UBE3A: 50%–60% relative to WT in the cortex and hippocampus, 99% in the striatum, and 44.6% in the cerebellum (Figure 4, C–F). Moreover, both the nuclear (Iso3) and cytosolic (Iso2) isoforms of UBE3A were reinstated in ASO-treated AS mice (Figure 4, C–F) (25).

We previously observed that in contrast to most behavioral phenotypes, there is no critical period for restoring hippocampal plasticity upon reinstatement of *Ube3a* gene expression. Moreover, we found that the critical period for rescuing the audiogenic seizure phenotype closes around the first 3 weeks after birth (ref. 16 and Y Elgersma, unpublished observation). Hence, we assessed both measures in the P21 injected mice. Three weeks after P21 injection, mice were tested for susceptibility to audiogenic seizures. All the PBS-injected AS mice were susceptible to induction of an audiogenic seizure, whereas none of the PBS-WT mice and ASO-treated AS mice showed seizures (Figure 4G).

At 8 weeks of age, we investigated hippocampal synaptic plasticity in these mice. A significant defect in LTP was observed in AS mice treated with PBS. ASO-treated AS mice, injected at P21, showed some improvement of LTP, but this was not significantly different from PBS-treated AS mice or from WT mice (Figure 4H).

## Discussion

Functional loss of UBE3A expression in neurons causes AS. Hence, regardless of the type of AS mutation, bringing back UBE3A protein could be a powerful disease-modifying treatment for AS. In this study, we demonstrate a very effective and specific action of a PS-LNA modified ASO gapmer targeting the *Ube3a-ATS* transcript to restore *Ube3a* expression in mouse neurons. Since the optimal treatment window in mice closes within the first 3 weeks of age (refs. 16–18 and Y Elgersma, unpublished observation), injections were performed at P1 or P21. ICV injection of ASO in *Ube3a^m–/p+^* mice at P1 or P21 resulted in a wide distribution of UBE3A throughout the brain. However, in the hippocampus of both P1 and P21 ASO-treated AS mice, lower UBE3A reinstatement was observed in the dentate gyrus, possibly due to neurogenesis, which occurs from embryonic development until adulthood, leading to dilution of the ASO (Figure 2A and Figure 4B) (20, 21, 26).

UBE3A reinstatement appears not to be limited to a specific cellular subtype as shown by immunofluorescence staining. In addition, we showed that the 2 highly conserved UBE3A isoforms, UBE3A-Iso3 (nuclear) and UBE3A-Iso2 (cytosolic), were both reinstated by ASO treatment at a similar ratio as WT mice (25). UBE3A was highly expressed (around 70% the level of UBE3A in WT) in the cortex and hippocampus 2 weeks after the injection and was still detectable in the brain 4–5 weeks later at 45%–50% the level of expression in WT mice, indicating an effective reinstatement of functional UBE3A. Measurement of UBE3A levels in the brain until 9 weeks of age indicated that at least until 9 weeks after ICV injection, the ASO was still partially functional in the brain.

Despite the high efficacy of ASO-induced UBE3A reinstatement at the molecular level, rescue of AS neurocognitive phenotypes was only partially achieved. The open field, forced swim test, and sensitivity to audiogenic seizure phenotypes were fully rescued. In addition, we observed improvement in the reverse rotarod performance. No effect was observed on the marble burying and nest building phenotypes. We have previously shown that the critical period for rescuing the marble burying and nest building phenotypes lies around birth (17). A lack of a rescue in the present work may be due to the P1 ASO injection acting too late. It is also possible that the amount of UBE3A needed to rescue these phenotypes is higher than we currently achieved or that more time is needed for the underlying circuits to adapt. Concerning the performance on the rotarod, although we previously showed that the critical period for rescuing this phenotype is longer than 3 weeks of age (16, 23), the lack of a typical learning curve, observed in both WT and AS mice, might have contributed to the lack of a clear ASO-mediated rescue in the initial rotarod test (16, 23). The lack of a good learning curve may have been caused by the compressed testing battery we adopted for this study, the single housing, or the younger age of the mice (6 weeks). These modifications to the standard battery protocol were adopted to ensure maximal levels of UBE3A during testing, and mice were single-housed during testing to assess nest building behavior. In order to evaluate motor coordination deficit more reliably, we generated a new cohort of mice, where the stress factor, which might have contributed to poor performance of the mice on the rotarod, was eliminated by testing these mice exclusively on the reverse rotarod and having them group-housed and well handled for 3 weeks. Further, the reverse rotarod task is more challenging; therefore, a difference in performance between the ASO and PBS-treated AS mice should be easier to detect (17). Accordingly, the new cohort showed a clear difference between AS and WT mice, and a good learning curve was observed for all groups. Moreover, motor coordination deficiency was significantly improved but not fully rescued in ASO-treated AS mice.

Regarding characteristic anatomical features of AS mice, even though body weight has repeatedly been shown to be increased in AS mice, we did not observe a difference between any of the 3 groups. This result is probably due to the young age (7 weeks) at which body weight was evaluated because studies that show obesity in AS mice report body weight at least from 8 weeks of age (23). A trend of improvement in brain weight was observed in 7-week-old ASO-treated AS mice. We speculate that a significant rescue of decreased brain weight might have been observed if the groups’ size had been larger and the mice had been older, as a rescue of this phenotype has been shown in a Cas9 gene therapy study where brain weight was measured at 40 weeks of age (27).

Importantly, we were able to fully rescue the sensitivity to audiogenic seizures in AS mice upon both P1 and P21 ASO injection. This result was notable, as we previously failed to rescue epilepsy upon Cre-mediated activation of the *Ube3a* gene initiated at P21 (16). Possibly, the action of ASO-mediated gene reactivation is faster acting than the tamoxifen-induced gene reactivation. ASOs are readily taken up by the cells, which leads to a rapid degradation of the *Ube3a-ATS*. In addition, in our previous study, we needed to reinstate UBE3A at P21 through repeated injections of tamoxifen over the course of 7 days, which could be too late for effective UBE3A reinstatement within the critical period (16). Alternatively, although the total amount of UBE3A expression achieved by tamoxifen injection compared with ASO injection was comparable on Western blot analysis, Cre-mediated expression of the *Ube3a* gene in each cell was either 0% or 100%, whereas ASO-induced UBE3A expression may affect more cells but yield lower UBE3A levels per cell. The rescue of other established AS phenotypes was not investigated, since we have previously shown that only the rotarod can be rescued upon UBE3A reinstatement at juvenile age (16).

Given the absence of clear cognitive phenotypes in *Ube3a* mice, we used LTP as a proxy to look at hippocampal plasticity. Our previous findings indicated that there is no critical period for reversing the LTP deficit (16). The LTP phenotype in P1 ASO-treated AS mice was comparable to that of WT mice, and in P21 treated mice it was not significantly different from either WT or AS mice injected with PBS. The absence of a significant LTP phenotype in P1 PBS-treated AS mice may be caused by the different background or age of the mice used in this study. Alternatively, we cannot rule out that the LTP levels of PBS-treated AS mice were affected by the audiogenic seizure that this subgroup had experienced. Last, we cannot rule out that the reduced expression of UBE3A in the dentate gyrus affected the LTP phenotype.

In conclusion, our results show that the ASO treatment was very effective at a molecular level and partially successful for the rescue of AS phenotypes in mice. Therefore, we believe this is a highly promising treatment to test in clinical trials. In particular, the ability to restore epilepsy at either P1 or P21 is very promising. Our study has 3 major translational limitations. First, the ASO we tested is specific for the mouse sequence of the *Ube3a-ATS* and cannot be used for clinical studies. Second, mouse phenotypes such as marble burying, nest building, and forced swim test have limited translational value as the underlying brain circuits are not fully understood. Important neurocognitive deficits, such as intellectual disability and sleep and speech impairments, which were not assessed in this study, might be improved in humans upon ASO treatment. Last, we do not know how the critical treatment window in humans compares to mice. Upcoming clinical trials in humans will need to clarify this.

Although we have previously shown that the presence of UBE3A is in particular important in the first postnatal weeks in mice, continuous presence of UBE3A is needed for certain behavioral tasks, such as the forced swim test (18). Hence, a limitation of ASO treatment for AS is that the treatment has to be continued, possibly lifelong. As we observed in this study, UBE3A expression decreased over time; thus, multiple injections are needed. It has recently been shown that AAV-mediated approaches can also be used to target the *Ube3a-ATS* (27). Such a strategy would be a major step forward, but such an irreversible treatment needs to ensure no off-site targeting as well as improved vectors to enable target engagement throughout the entire brain. On the other hand, ASOs are transient, reversible, and titratable, and the burden of repetitive lumbar intrathecal injections might be reduced by using implantable devices or replaced by Ommaya (or Rickham) reservoirs, which enable delivery of drugs directly into the ventricles.

## Methods

### ASO design and in vitro screening.

ASOs (*n* = 192) were designed and synthesized as previously described (28). All ASOs were complementary to the minus strand of chromosome 7 in a 34 kb region (chr7: 59307929 –59341825, GRCm38) downstream of the *Ube3a* gene, which is partly overlapping with the annotated transcript *Snhg14* (*Ube3a-ATS*). ASOs were screened at 2 concentrations (0.05 μM and 2 μM), for their ability to knock down the *Ube3a-ATS*, in embryonic (E14–E15) mouse primary cortical cultures. The potency of the most efficacious ASOs was determined in primary cortical cultures prepared from *Ube3a^m–/p+^* P1 pups by generating concentration response curves measuring the *Ube3a-ATS* and *Ube3a* mRNA by quantitative PCR. ASO RTR26266 was identified as the most effective compound. ASO RTR26266 has a fully modified PS backbone and LNA modifications at the 5′ and 3′ extremities. The nucleotide sequence of ASO RTR26266 is TCCaacttaataaCCT, where capital letters are LNA modifications (all LNA-C nucleotides contain the 5-methylcytosine modification) and lowercase letters are DNA.

### Mouse husbandry and breeding.

In this study, we used *Ube3a^m−/p+^* mice (*Ube3a^tm1Alb^*; MGI 2181811; ref. 19) for all experiments. *Ube3a^tm1Alb^* mice were maintained (>40 generations) in the 129S2 background (full name: 129S2/SvPasCrl) by crossing male *Ube3a^m+/p−^* mice with female 129S2 WT mice. For behavioral experiments, female *Ube3a^m+/p−^* mice were crossed with male C57BL/6J mice to generate *Ube3a^m−/p+^* (AS) mice and their WT littermates in an F1 hybrid 129S2-C57BL/6J background. For epilepsy experiments, female *Ube3a^m+/p−^* mice were crossed with male 129S2 mice to generate *Ube3a^m−/p+^* (AS) mice and their WT littermates in a 129S2 background. Mice were housed in individually ventilated cages (IVC; 1145T cages from Techniplast) in a barrier facility. Mice were genotyped when they were 4–7 days old and regenotyped at the end of the experiment. All animals were provided with mouse chow [801727CRM(P) from Special Dietary Service] and water ad libitum. Mice were kept at 22 ± 2°C with a 12-hour light/12-hour dark cycle. P1 injected mice were single-caged 1 week prior to the start of the behavioral battery (at 5 weeks of age), whereas the mice that were injected at P21 were single-caged from that point on. The mice used for the reverse rotarod task were group-housed with 2 to 4 animals of the same sex per cage.

### ICV injections of ASO.

ASOs were diluted to a final concentration of 22 μg/μL in PBS and 0.3% Fast Green. P1 mice were anesthetized on ice and placed in a stereotaxic frame (David Kopf Instruments). A glass pipet (0.5–0.7 μm tip) containing either ASO or PBS was attached to a 25 μL syringe (Hamilton, 1702N 25 μL micro syringe) and placed at the middle of a line defined between the right eye and the lambda intersection of the skull. The needle was lowered into the lateral ventricle to a depth of approximately 3 mm to inject 1 μL of PBS or ASO (22 μg) at a flow rate of 0.5 μL/min using a CMA 400 Syringe Pump (Harvard Apparatus). After the injection, the pups recovered on a heating pad and under a heat lamp before being placed back with the mother.

For ICV injections at P21, mice were anesthetized with 4% isoflurane and placed in a stereotaxic frame (David Kopf Instruments). During the whole surgery, the mice were kept anesthetized with 1.5% isoflurane. After exposing the skull, a glass needle (0.5–0.7 μm tip) was placed 0.5 mm posterior and 1.0 mm lateral to the bregma and lowered to a depth of 1.5 mm from the meninges. Injection of 3 μL of ASO at a final concentration of 30.3 μg/μL or PBS was carried out at a flow rate of approximately 0.5 μL/min using a CMA 400 Syringe Pump. The needle was left in the brain for 5 minutes after the injection, then slowly retracted, and the skin incision was sutured. After the surgery the mice were individually caged and left to recover next to a heating lamp.

### Behavioral battery.

At either P1 or P21, AS mice and WT mice, both male and female, received a single dose of ASO or PBS. At 6 weeks of age, a battery of behavioral tests was performed. Before the start of the behavioral tests, the mice were handled for 2 weeks. All behavioral experiments were performed during the light period of the light/dark circadian cycle by an experimenter blind to genotype and treatment. The sample size required for each test was calculated based on genotype and treatment, following the power analysis as previously published (23). Behavioral tests were always run in the following order: day 1: open field; day 2: marble burying; accelerating rotarod test from day 2 to day 6 at the same time every day; nest building test from day 2 to day 6 (weight of the nest was assessed at the same hour every day); forced swim test on day 6 (after the rotarod) and day 7. All battery tests were performed as previously described (23). In brief, for the open field test, mice were individually placed in a 110 cm diameter circular arena and given 10 minutes to explore the area. An infrared camera (Noldus Wageningen) connected to EthoVision software (Noldus Wageningen) was used to record the total distance moved by each mouse in the open arena. For marble burying, mice were individually placed in a cage filled with 4 cm of bedding material (Lignocel Hygienic Animal Bedding, JRS) and 20 blue glass marbles arranged on top of the bedding material in a 5 × 4 grid. All mice were given access to the marbles for 30 minutes. Only the marbles covered for more than 50% by bedding were scored as buried. Motor performance was assessed using the accelerating rotarod (4–40 rpm, in 5 minutes; model 7650, Ugo Basile Biological Research Apparatus). Mice were tested in 2 trials per day with a 40-minute intertrial interval. For each day, we calculated the average time spent on the rotarod or the time until the mouse made 3 consecutive passive rotations on the rotarod (latency indicated in seconds). Maximum duration of a trial was 5 minutes. For the reverse rotarod, a similar method was used, using a modified rotarod that minimizes the possibility of mice to turn around, and we calculated the average time spent on the rotarod or the time until the mouse made 3 consecutive passive rotations or until the mice managed to turn around on the rotarod (17). Mice used for the reverse rotarod were prehandled for 3 weeks to minimize anxiety and jumping behavior. To assess nest building, 11 g (11 ± 1) of compressed, extrathick blot filter paper (Bio-Rad) was placed in the cage. Every day the amount of unused nest material was noted. In the forced swim test, mice were placed for 6 minutes in a cylindrical transparent tank (27 cm high and 18 cm diameter) filled with water (26 ± 1°C) 15 cm deep. At the beginning of the test, each mouse was left to acclimatize in the cylinder for 2 minutes. The floating time was determined by scoring manually (stopwatch) for 4 minutes the amount of time (seconds) the mouse was not moving.

To test susceptibility to audiogenic seizures, a maximum of 3 same-sex littermates were placed in a Makrolon (polycarbonate) cage (50 × 26 × 18 cm). Audiogenic seizures were induced by vigorously scraping scissors across the metal grating of the cage lid (approximately a 100 dB sound). This noise was induced for 20 seconds or less if a (tonic-clonic) seizure developed before that time. Seizures were noted by visual observation.

### Electrophysiology.

One week after the epilepsy test, *Ube3a^m–/p+^* mice were sacrificed, and hippocampal slices of 400 μm were obtained using a vibratome. Extracellular field recordings were obtained in a submerged recording chamber perfused continuously with artificial cerebrospinal fluid. LTP was evoked by using the 10-theta burst protocol performed at two-thirds of the maximum fEPSP.

### Immunohistochemistry.

Mice were perfused transcardially with 4% paraformaldehyde after being euthanized with pentobarbital. Brain tissue was dehydrated in 10% sucrose overnight and embedded in a solution with 12% gelatin and 10% sucrose. Sagittal sections of 40 μm were cut using a microtome (SM2000R; Leica Microsystems). Primary antibody staining was performed using anti-UBE3A (mouse E6AP clone 3E5, MilliporeSigma, SAB1404508) at a concentration of 1:750 and anti-parvalbumin (rabbit, Swant, PV27) at a concentration of 1:5000. Secondary antibody staining was performed using donkey anti-mouse 488 (Jackson ImmunoResearch, 715545150, 1:200) and donkey anti-rabbit Cy3 (Jackson ImmunoResearch, 711165152, 1:200), and it was followed by DAPI staining for 10 minutes. Tissue slices were mounted on 24 *×* 40 mm coverslips. Images were taken using LSM700 Zeiss Confocal Laser Scanning Microscope and analyzed using Fiji.

### Immunoblotting.

Mice were euthanized by cervical dislocation. The cortex, hippocampus, striatum, and cerebellum were extracted. Brain sections were homogenized and lysed in 2× Laemmli buffer, with 1% protease inhibitor, by sonicating 3 times for 3 seconds. Protein concentration was determined using the BCA protein assay kit (Thermo Fisher Scientific, 23225). Twenty micrograms of proteins were separated on a precast 4–12% Criterion XT Bis-Tris gel (Bio-Rad) and transferred to a nitrocellulose membrane using TurboBlot (Bio-Rad). The following antibodies were used: anti-UBE3A (anti-E6AP, MilliporeSigma, catalog E8655, 1:1000) and anti-actin (Chemicon, MAB1501R, 1:20,000). The next day, the membranes were probed with secondary goat anti-mouse antibody (LI-COR Biosciences, IRDye 800CW, 926-32210, 1:15,000). The membranes were scanned using Odyssey CLx (LI-COR Biosciences) and quantified using the Odyssey 3.0 software.

### Half-life of ASO-induced reinstatement.

Normalized UBE3A levels determined in the cortex and hippocampus at different time points after P1 injection were used to construct a time curve (Figure 2, C and D). Interpolation of the equation obtained from the line of best fit was used to calculate the time for a reduction of 50% in UBE3A protein levels.

### Statistics.

Data were analyzed using Excel 2018 (Microsoft) and GraphPad Prism software, and values of *P* ≤ 0.05 were considered significant. Western blot was analyzed using a 1-way ANOVA, 2 sided, with the variable genotype plus treatment as independent variable and followed by Tukey’s post hoc test. Before performing any of the following statistical tests, the samples were analyzed for normal distribution. Open field, marble burying, and brain weight data were analyzed using a 1-way ANOVA, 2 sided, with the variable genotype plus treatment as independent variable and followed by Bonferroni’s or Tukey’s post hoc test; forced swim test data were analyzed using the Kruskal-Wallis test, 2 sided, with the variable genotype plus treatment as independent variable and followed by Dunn’s post hoc test. Both the accelerating and reverse rotarod and nest building were measured with a 2-way repeated-measure ANOVA, 2 sided, followed by Tukey’s post hoc test, with the variables genotype plus treatment and time as independent variables. Fisher exact test, 2 sided, was used to evaluate the correlation between treatment and seizure susceptibility. LTP was analyzed using a 2-way ANOVA, 2 sided, with genotype plus treatment as independent variable and followed by Bonferroni’s post hoc test. All values are represented as means ± SEM. *P* values represent the significance level for the parameter and are displayed as asterisks in the figures and not shown if *P* > 0.05 (Supplemental Table 1).

### Study approval.

All animal experiments were conducted in accordance with the European Commission Council Directive 2010/63/EU, and we obtained national and local approval under CCD license AVD101002016791 (Centrale Commissie Dierproeven, the Hague, the Netherlands).

## Author contributions

YE, MTM, MCH, T Kremer, and EJM conceived and designed the study. SVR and KDE designed and tested the ASO in vitro. CM, IW, EJM, and T Kakunuri carried out the behavioral testing, and CM, IW, EJM, T Kakunuri, and ASEVDS performed the molecular and immunostaining experiments. CM performed the statistical analysis. CM, EJM, and YE analyzed and interpreted the data. CM and EJM made the figures. CM, EJM, and YE wrote the first manuscript. All authors edited and approved the final manuscript.

## Supplementary Material

Supplemental data

## Version 1 08/09/2021

Electronic publication
