# Supplementary material for: Antisense oligonucleotide treatment rescues UBE3A expression and multiple phenotypes of an Angelman syndrome mouse model
Source: JCI Insight. 2021 Aug 9;6(15):e145991. doi: 10.1172/jci.insight.145991 (PMC8410092; doi:10.1172/jci.insight.145991)
Supplement: Supplemental data [file jciinsight-6-145991-s305.pdf]

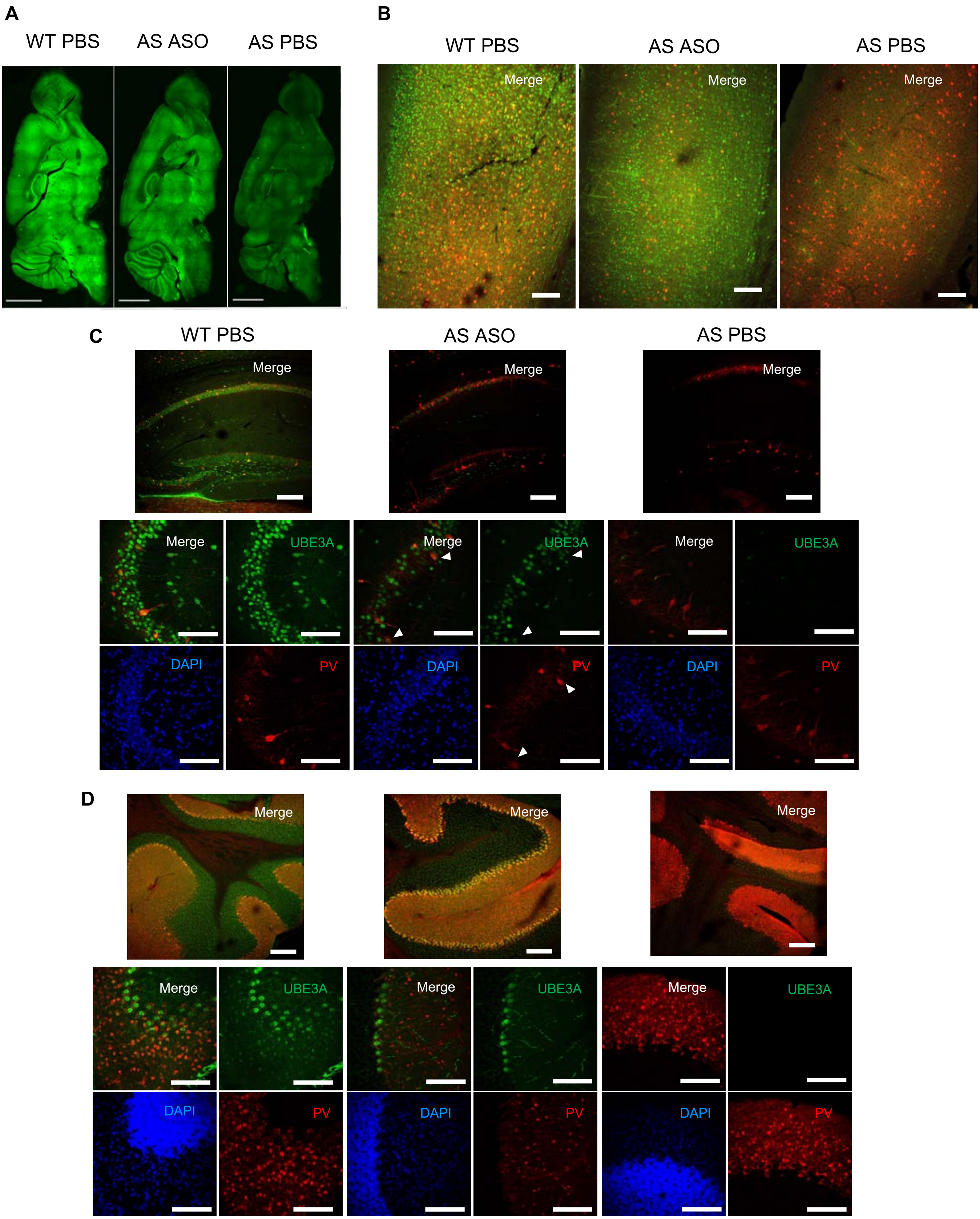

Supplemental Figure 1: Reinstatement of UBE3A in AS mice injected with the ASO at P1 is not limited to glutamatergic neurons. Four weeks post-P1 injection the brains (n=2) of WT-PBS, AS-ASO and AS-PBS injected mice were stained for UBE3A (green). (A) The brain of AS mice treated with ASO shows wide spread UBE3A reinstatement compared to the untreated AS brain. (B-D) the cortex (B) hippocampus (C) and cerebellum (D) of WT-PBS, AS-ASO and AS-PBS mice were stained for UBE3A (green), for parvalbumin (PV) (red) and counterstained for DAPI (blue). Scale bars, 2000 µm (A), 200 µm (B), top panel 200 µm, lower two panels 100 µm (C-D). Arrows point at cells positive for both parvalbumin and UBE3A.

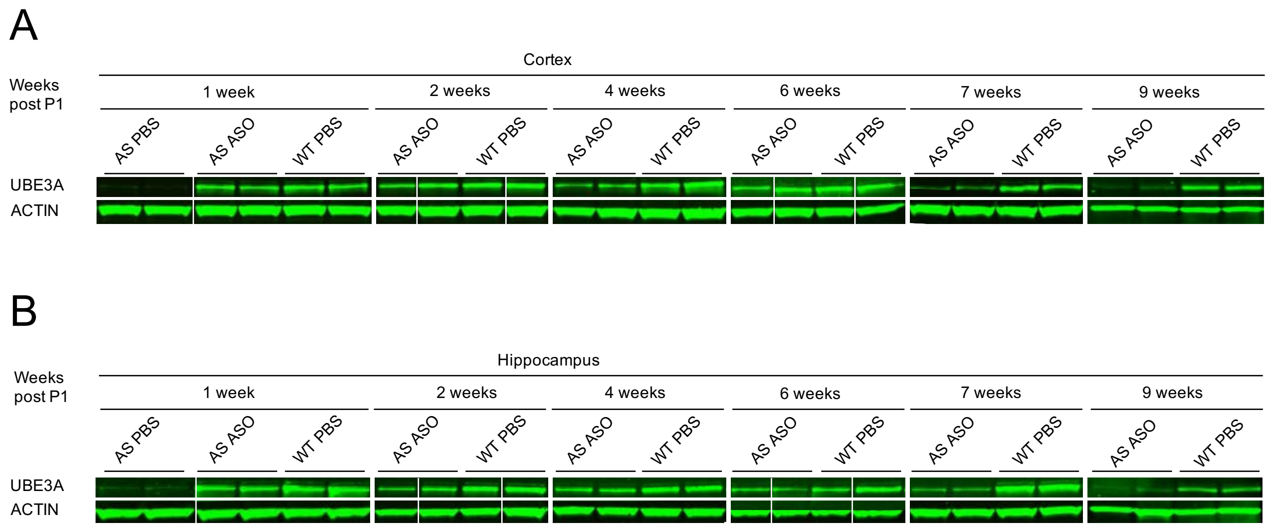

Supplemental Figure 2: ASO successfully restores UBE3A in the brain of P1 injected AS mice. Brains of ASO treated AS mice were used for Western Blot analysis at different time points: 1 week after P1 injection (n=7), 2 (n=3), 4 (n=3), 6 (n=2) and 7 (n=4) weeks after P1 injection. AS-PBS (n=2) and WT (n=3) littermates were used as controls. The thin white vertical lines separating bands indicate noncontiguous lanes that were run on the same gel. UBE3A can be detected at 100kDa and actin, used as loading control, at 45kDa.

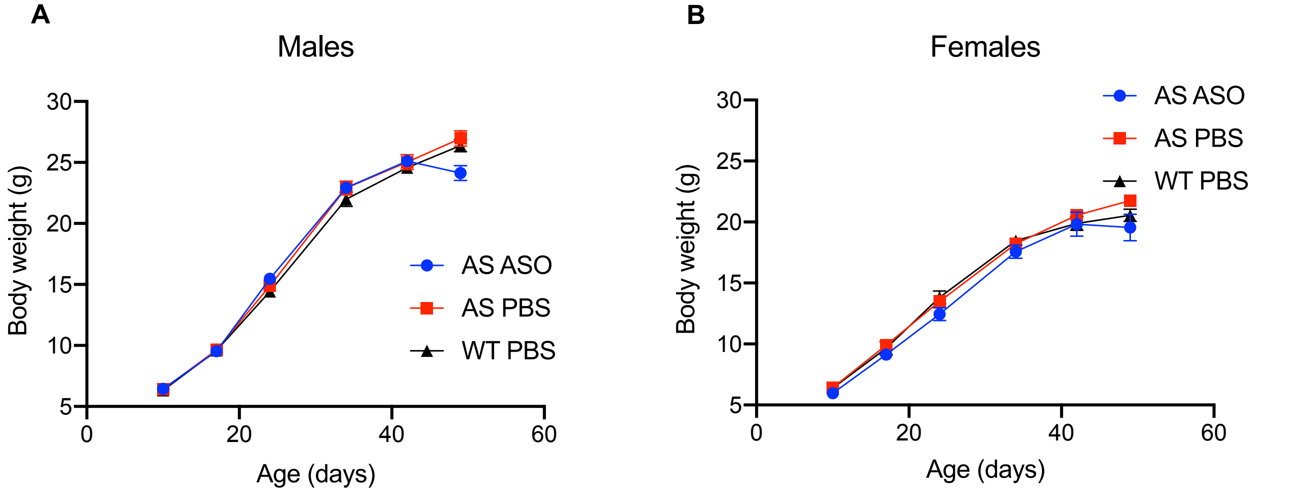

Supplemental Figure 3: Body weight does not change among groups. The body weight of AS ASO, WT PBS and AS PBS treated mice is noted at different ages, over the course of 49 days. A. Body weight of males only (AS ASO :10 and 17 days n=10; 24, 34 and 42 days n=9; 49d n= 4. WT PBS: 10, 24 and 42 days n=13; 17d n=12; 34 days n=10; 49d n= 4. AS PBS: 10, 24, and 42 days n=11; 17d n=7; 34d n=10; 49d n= 4). B. Body weight of females only (AS ASO: 10d n=9; 17, 24, 34, and 42 days n=10; 49d n=4. WT PBS: 10, 24 and 42 days n=7; 17d n=5; 34d n=4; 49d n=5. AS PBS:10, 24, and 42 days n=7;17 and 34 days n=4; 49d n=5). Data is represented as means ± SEM.

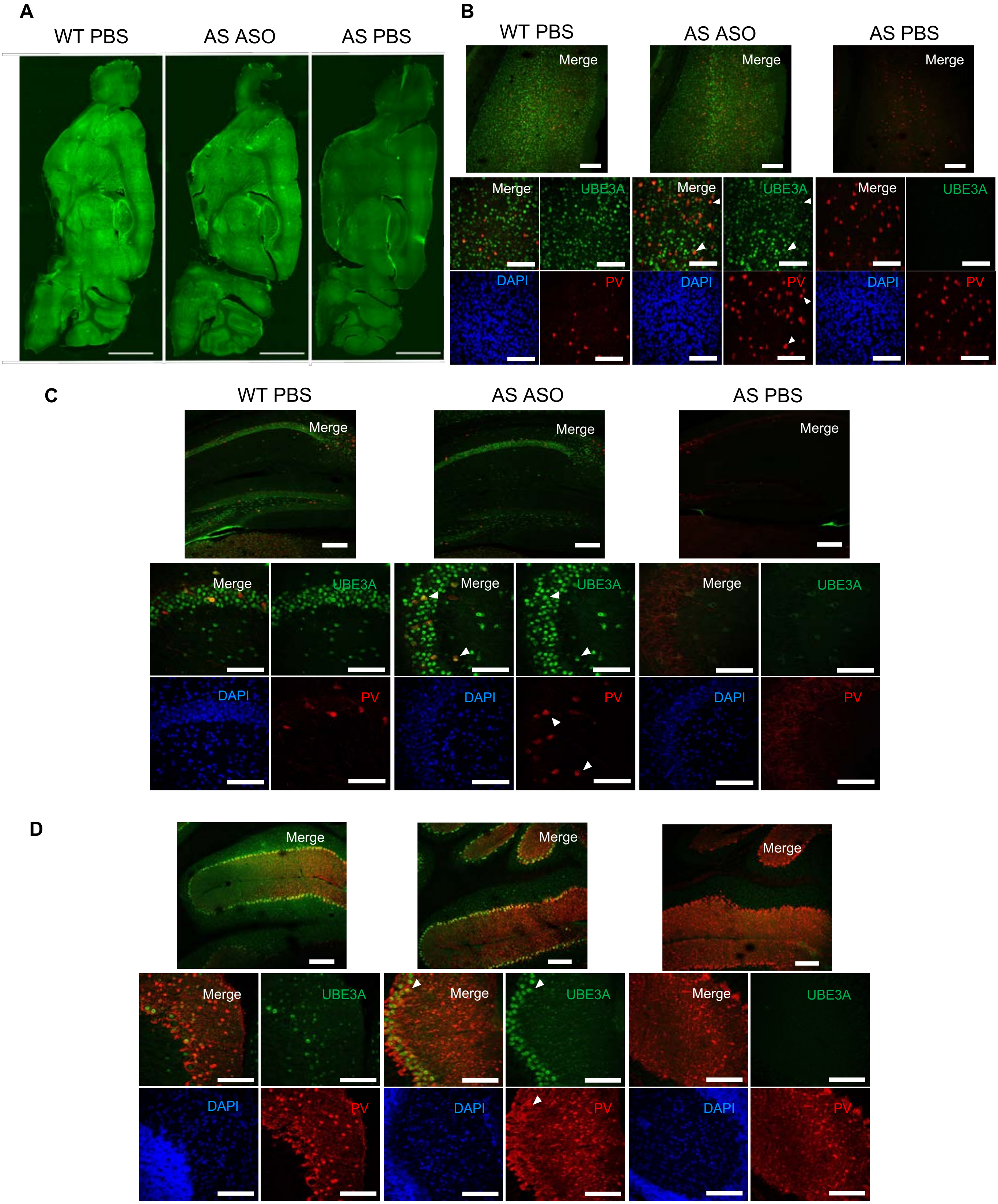

Supplemental Figure 4: Reinstatement of UBE3A in ASO treated AS mice at P21 is not limited to glutamatergic neurons. Three weeks post-P21 injection the brains (n=2) of WT-PBS, AS-ASO and AS-PBS injected mice were stained for UBE3A (green). (A) The brain of AS mice treated with ASO shows wide spread UBE3A reinstatement compared to the untreated AS brain. (B-D) the cortex (B) hippocampus (C) and cerebellum (D) of WT-PBS, AS-ASO and AS-PBS mice were stained for UBE3A (green), for parvalbumin (PV) (red) and counterstained for DAPI (blue). Scale bars, 2000 µm (A), top panel 200 µm, lower two panels 100 µm (B-D). Arrows point at cells positive for both parvalbumin and UBE3A.

Supplemental Table 1: Summary of the statistical tests.

	Behavioral test	Dependent variable	Normality Test	Sample N	Test statistic	p value	Test statistic	Directionality	Independent variable	Test statistic value (df)	p value F test	Post-hoc test	Comparison	Adjusted p-value
P1 injected mice	rotarod	latency to fall (s)	Shapiro-Wilk test	AS ASO =16	W: 0.950	0.735	2-way repeated measure ANOVA	2-sided	Genotype-Treatment	F (2,43)=14.339	**<0.0001**	Tukey	AS ASO vs. AS PBS	0.100
													AS ASO vs. WT PBS	**0.005**
													AS PBS vs. WT PBS	**<0.0001**
				AS PBS =16	W: 0.971	0.881		2-sided	Time	F (2.9, 124.7) = 0.799	0.49			
				WT PBS =14	W: 0.946	0.708		2-sided	Genotype-Treatment*Time	F (5.8, 124.7) = 1.375	0.23			
	open field	distance moved (cm)	D'Agostino & Pearson	AS ASO =16	K2: 2.893	0.235	Univariate ANOVA	2-sided	Genotype-Treatment	F (2, 43) = 8.824	**<0.001**	Bonferroni	AS ASO vs. AS PBS	**0.003**
				AS PBS =16	K2: 1.723	0.422							AS ASO vs. WT PBS	>0.999
				WT PBS =14	K2: 0.168	0.919							AS PBS vs. WT PBS	**0.002**
	marble burying	% buried marbles	D'Agostino & Pearson	AS ASO =16	K2: 3.519	0.172	Univariate ANOVA	2-sided	Genotype-Treatment	F (2, 42) = 24.05	**<0.0001**	Bonferroni	AS ASO vs. AS PBS	0.66
				AS PBS =16	K2: 4.971	0.083							AS ASO vs. WT PBS	**<0.0001**
				WT PBS =15	K2: 3.935	0.140							AS PBS vs. WT PBS	**<0.0001**
	nest building	used nesting material (%)	Shapiro-Wilk test	AS ASO =16	W: 0.966	p=0.85	2-way repeated measure ANOVA	2-sided	Genotype-Treatment	F (2, 42) = 16.64	**<0.0001**	Tukey	AS ASO vs. AS PBS	0.783
													AS ASO vs. WT PBS	**<0.0001**
													AS PBS vs. WT PBS	**<0.0001**
				AS PBS =16	W: 0.977	p=0.919		2-sided	Time	F (4, 168) = 150.4	**<0.0001**			
				WT PBS =14	W: 0.981	p=0.939		2-sided	Genotype-Treatment*Time	F (8, 168) = 9.795	**<0.0001**			
	forced swim test	floating time (%)	D'Agostino & Pearson	AS ASO =16	K2: 8.168	**0.017**	kruskall-wallis	2-sided	Genotype-Treatment	H(2,43)=15.94	**0.0003**	Dunn's	AS ASO vs. AS PBS	**0.002**
				AS PBS =16	K2: 31.86	**<0.0001**							AS ASO vs. WT PBS	>0.999
				WT PBS =14	K2: 3.393	0.183							AS PBS vs. WT PBS	**0.002**
	reverse rotarod	latency to fall (s)		AS ASO =17			2-way repeated measure ANOVA	2-sided	Genotype-Treatment	F (2, 63) = 15.48	**<0.0001**	Tukey	AS ASO vs. AS PBS	**0.038**
													AS ASO vs. WT PBS	**0.0321**
													AS PBS vs. WT PBS	**<0.0001**
				AS PBS =30				2-sided	Time	F (4, 252) = 90.00	**<0.0001**			
				WT PBS =19				2-sided	Genotype-Treatment*Time	F (8, 252) = 3.826	**0.0003**			
	brain weight	weight (g)	Shapiro-Wilk test	AS ASO =8	W: 0.902	0.3014	Univariate ANOVA	2-sided	Genotype-Treatment	F (2, 20) = 11.69	**0.0004**	Tukey	AS ASO vs. AS PBS	0.1062
				AS PBS =7	W: 0.918	0.4564							AS ASO vs. WT PBS	**0.0311**
				WT PBS =8	W: 0.942	0.6293							AS PBS vs. WT PBS	**0.0003**
	Induced Audiogenic Seizures	Seizures (%)		AS ASO =10			Fisher Test	2-sided	Treatment	AS PBS vs AS ASO	**0.0001**			
				AS PBS =6										
				WT PBS =7										
	LTP	fEPSP slope (% of baseline)	-	AS ASO = 16/8 (slice per mouse)			Two-way ANOVA	2-sided	Genotype-Treatment	F (2, 39) = 3.56	**0.038**	Bonferroni	AS ASO vs. AS PBS	0.065

				AS PBS = 13/16 (slice per mouse) WT PBS = 13/6 (slice per mouse)									AS ASO vs. WT PBS	1.0
													AS PBS vs. WT PBS	0.089

	**Behavioral test**	**Dependent variable**	**Normality Test**	**Sample N**	**Test statistic**	**p value**	**Test statistic**	**Directionality**	**Independent variable**	**Test statistic value (df)**	**p value F test**	**Post-hoc test**	**Comparison**	**Adjusted p-value**
P21 injected mice	Induced Audiogenic Seizures	Seizures (%)		AS ASO =13 AS PBS =10 WT PBS =9			Fisher Test	2-sided	Treatment	AS PBS vs AS ASO	**<0.0001**			
	LTP	fEPSP slope (% of baseline)	-	AS ASO = 27/7 (slice per mouse) AS PBS = 26/5 (slice per mouse) WT PBS = 23/4 (slice per mouse)			Two-way ANOVA	2-sided	Genotype-Treatment	F (2, 73) = 3.56	**0.018**	Bonferroni	AS ASO vs. AS PBS	0.471
													AS ASO vs. WT PBS	0.371
													AS PBS vs. WT PBS	**0.014**

	**Western Blot**	**Dependent variable**	**Normality Test**	**Sample N**	**Test statistic**	**Directionality**	**Independent variable**	**Test statistic value (df)**	**p value F test**	**Post-hoc test**	**Comparison**	**Adjusted p-value**
P21 injected mice	Cortex	UBE3A (%)	N too small	AS ASO = 3 AS PBS = 3 WT PBS = 3	Univariate ANOVA	2-sided	Genotype-Treatment	F (2, 6) = 21.38	**0.002**	Tukey	AS ASO vs. AS PBS	**0,028**
											AS ASO vs. WT PBS	0,055
											AS PBS vs. WT PBS	**0,002**
	Hippocampus			AS ASO = 3 AS PBS = 3 WT PBS = 3				F (2, 6) = 75.53	**<0.0001**	Tukey	AS ASO vs. AS PBS	**0,003**
											AS ASO vs. WT PBS	**0,002**
											AS PBS vs. WT PBS	**<0.0001**
	Striatum			AS ASO = 3 AS PBS = 3 WT PBS = 3				F (2, 6) = 9.38	**<0.0002**	Tukey	AS ASO vs. AS PBS	**0,024**
											AS ASO vs. WT PBS	0,988
											AS PBS vs. WT PBS	**0,020**
	Cerebellum			AS ASO = 2 AS PBS = 2 WT PBS = 2				F (2, 4) = 107.2	**0.0003**	Tukey	AS ASO vs. AS PBS	**0,011**
											AS ASO vs. WT PBS	**0,001**
											AS PBS vs. WT PBS	**0,0003**
